# Analysis of tuberculosis surveillance data in Oyo State, Nigeria, 2011-2014

**DOI:** 10.11604/pamj.supp.2018.30.1.15273

**Published:** 2018-05-12

**Authors:** Oyindamola Bidemi Yusuf, Junaidu Kabir, Calbeth Chika Odinaka Alaribe

**Affiliations:** 1 Department of Epidemiology and Medical Statistics, Faculty of Public Health, University of Ibadan, Nigeria; 2 Department of Veterinary Public Health and Preventive Medicine, Ahmadu Bello University, Zaria, Nigeria; 3 Rollins School of Public Health, Emory University, Atlanta, USA

**Keywords:** Data analysis, tuberculosis, surveillance, Nigeria

## Abstract

The role of surveillance in tuberculosis (TB) management and control is imperative to the eradication of the disease. Training of TB focal persons, TB program officers and medical officers involved in data management will help to improve the quality of surveillance data. This case study was developed using data extracted from the Oyo state Integrated Disease Surveillance and Response (IDSR) database on TB from January 2011 to December 2014. The case study aims to evaluate surveillance data with the overall goal of improving data quality for TB control. The training will describe the requirements of routine surveillance as well as procedures for determining the burden of TB and the evaluation of some of the key indices for surveillance data quality. The participants must have prior knowledge of how to analyze and manage data and should be able to complete the exercises in approximately three hours.

## How to use this Study

**General instructions:** for the purpose of this case study one instructor per 10 participants will be required that will moderate the sessions involving 8-20 participants in a conference or similar room. Participants should sit facing each other while the instructors utilize approaches that provide an opportunity for all participants to contribute. The facilitator asks questions directed at named participants while answers follow discussions involving all or small groups of participants. Time is also given to participants to undertake calculations using laptop computers in generating answers.

**Audience:** medical officers, TB focal persons, and TB program officers working for government at the local, state and national level, private medical practitioners involved in Directly Observed Therapy (DOT) implementation.

**Prerequisites:** previous lectures on principles of surveillance and data analysis and prior training in basic epidemiology and basic knowledge of MS Excel.

**Materials needed:** flip charts, white board with markers, calculators, laptop computer with Microsoft Excel.

**Level of training and associated public health activity:** intermediate.

**Time required:** 3 hours.

**Language: english.**

## Competing interest

The authors declare no competing interest.
